# Timed-averaged blood pressure showed a J-curve association with stroke in elderly chronic kidney disease patients

**DOI:** 10.1080/0886022X.2022.2131574

**Published:** 2022-10-12

**Authors:** Chen Fu, Dongliang Zhang, Haiping Chen, Hui Zhang

**Affiliations:** a Department of Nephrology, Beijing Jishuitan Hospital, Beijing, China; b Department of Geriatric Nephrology, Medical and Health Care Center, Beijing Friendship Hospital (affiliated with Capital Medical University), Beijing, China; c Department of Nephrology, Tianjin Hospital, Tianjin, China

**Keywords:** Chronic kidney disease, elderly population, stroke, blood pressure, J-curve

## Abstract

**Background:**

The risk factors for stroke in elderly patients with chronic kidney disease (CKD) are not well understood. This study aimed to explore the influence of systolic blood pressure (SBP) on the risk of stroke in a large cohort of elderly patients with stage 3–5 CKD.

**Methods:**

We retrospectively identified 665 patients hospitalized in Beijing Friendship Hospital from January 2000 to December 2021. Patients were followed up until the occurrence of stroke or death. Multivariate logistic regression analysis and Cox proportional hazard models were used to analyze the risk factors for stroke according to the presence or absence of CKD. The association between CKD and stroke was further evaluated regarding the role of SBP in the hypertensive population.

**Results:**

In individuals with CKD, a J-shaped relationship was observed between SBP levels and the risk of stroke. Participants with CKD and an SBP less than 125 mmHg had a significantly higher cumulative stroke survival rate than those whose SBP was between 125 and 139 mmHg. The cumulative stroke survival rate increased progressively for those with SBP higher than 140 mmHg. This J-shaped relationship was not found in patients without CKD.

**Conclusion:**

In elderly patients with CKD, those with the lowest BP are at increased risk for incident stroke. This phenomenon could be different from that in the general population.

## Introduction

Chronic kidney disease (CKD) is a major health care burden worldwide, particularly among elderly patients. In this population, the incidences of both dialysis-requiring end-stage kidney failure and early-stage CKD are on the rise [1–3]. Hypertension (HTN), especially isolated systolic hypertension, is a risk factor for the development of stroke in the general population. In addition, HTN is closely associated with CKD since HTN is a major cause of CKD, and CKD leads to a higher risk of HTN [4].

Stroke is now the second leading cause of death worldwide, following cardiovascular disease and cancer [5]. The risk of stroke doubles for every 20/10 mmHg increase in blood pressure (BP) over 115/75 mmHg in the general population [6]. Consequently, adequate BP control is imperative for reducing the risk of stroke. Multiple placebo-controlled clinical trials have found that antihypertensive medications, such as diuretics [7], β-blockers [8], calcium antagonists, and angiotensin-converting inhibitors [9,10], are effective in decreasing the risk of stroke among patients regardless of the race or the type of drug used. The Study on Cognition and Prognosis in the Elderly (SCOPE) [11], a large-scale randomized, parallel-group study that enrolled approximately 5000 patients from 15 countries, demonstrated that elderly patients with an initial BP of 160 to 179 over 90 to 99 mm Hg and a treatment-induced BP reduction of 20/10 mm Hg exhibited a 24% reduction in the risk of stroke. However, the literature does not show similar findings in patients with CKD. An observational study suggested that the risk of stroke and death increased significantly among CKD patients with systolic BP (SBP) ≤ 120 mmHg compared to that among those with SBP above 120 mmHg [12]. Furthermore, a J-shaped relationship between SBP and risk of stroke was identified, with an increased risk observed for patients with lower SBP [4,13,14]. Nonetheless, few studies have evaluated this phenomenon in elderly patients with early-stage CKD.

Elderly patients with CKD are different from their younger counterparts. Frequently, we cannot identify a primary cause of CKD in elderly individuals, particularly when the diagnosis is established only by a mildly reduced glomerular filtration rate (GFR). A consensus has not been reached regarding the diagnostic criteria for CKD in the elderly population. It still remains controversial whether an elderly person with a mild reduction in estimated GFR (eGFR) without evidence of any renal damage should be diagnosed with CKD [15]. However, several studies have shown that decreased eGFR and albuminuria (both alone and in combination) are associated with an increased risk of poor outcomes, including cardiovascular mortality and all-cause death, in the elderly population [16,17]. This suggests that the current definition of CKD in the elderly population could be reasonable, but the threshold for defining eGFR reduction in these patients is still undetermined.

Although SBP is a well-known risk factor for stroke in the general population [6], questions remain concerning the influence of BP measurement techniques. For example, the timing of BP measurements might require clarification. In addition, a history of hypertension is not necessarily associated with poor outcomes; SBP levels after treatment may be a better risk indicator. Finally, it is still unclear whether patients with initially higher BP have the same prognosis as those with initially lower BP if both groups of patients achieve target BP after treatment.

In this study, we aimed to investigate the relationship between SBP and the risk of stroke in a large cohort of elderly patients with CKD.

## Materials and methods

### Patient selection

**Figure 1.**
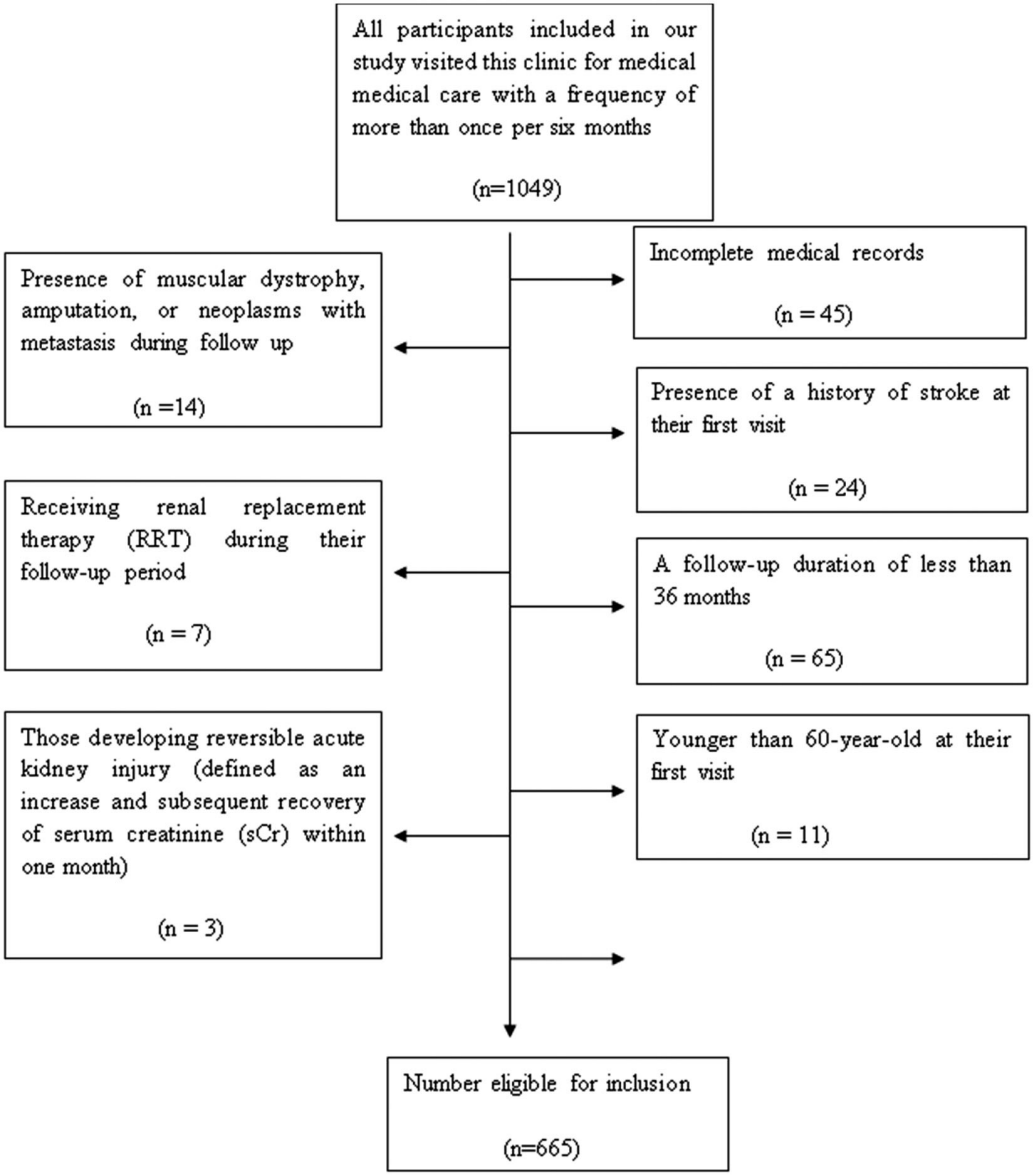
A flow diagram showing the application of exclusion criteria when enrolling participants in a study of systolic blood pressure and stroke in elderly individuals with chronic kidney disease.

We retrospectively reviewed the clinical data of patients hospitalized in the medical health care center of Beijing Friendship Hospital between January 1st, 2000 and December 31st, 2021. All participants (*n* = 1049) included in our study visited this clinic for medical care at a frequency of more than once every six months. Of these, 384 participants were excluded (Figure 1) due to incomplete medical records (*n* = 45); being younger than 60 years old at baseline (*n* = 11); having a follow-up time less than 36 months (*n* = 65); having a stroke at baseline (*n* = 24); receiving renal replacement therapy (RRT) in the follow-up period (*n* = 7); being in the period of reversible acute renal failure (a rapid reduction and recovery in creatine within a 1-mo time frame) (*n* = 3); having muscular dystrophy, amputation, or neoplasm metastasis (*n* = 14); and having no history of hypertension (*n* = 215). A total of 665 patients were enrolled in our study. The ethics committee of Beijing Friendship Hospital affiliated with Capital Medical University approved the study (ethics committee approval number: BJFH-EC/2010–035). Written informed consent was provided by all participants or their legal proxies. This study was registered with Chinese Clinical Trial Registry (No. chiCTR-ONRC-10000875).

We documented clinical information, including the clinical presentation at the first visit and other relevant information. Demographics and clinical data at baseline were collected from electronic medical records in the hospital. Demographic data, including age, sex, body height, body weight, past medical history, and substance use (smoking and alcohol consumption), were recorded at the time of their first assessment. Cigarette smoking and alcohol use were dichotomized as current use or nonuse. Diabetes was defined by the use of insulin or any oral hypoglycemic medications or a fasting glucose ≥ 7 mmol/l. The following clinical data were identified retrospectively: BP, usage of any medications, emergence of any new diseases, laboratory parameters (serum creatinine [sCr], uric acid [UA], glucose [Glu], total cholesterol [CHOL], high-density lipoprotein [HDL], low-density lipoprotein [LDL], triglycerides [TGs], urinalysis results), head computed tomography (CT) and magnetic resonance imaging (MRI).

### Standardization and monitoring of blood pressure

Patients were instructed to empty their bladder and avoid smoking, caffeine, and exercise for at least 30 min before measuring their BP. They were seated comfortably with their back supported and feet on the ground for 3 to 5 min before the readings. The patient and observer refrained from talking during the rest period and during BP measurement. Auscultatory semiautomatic was the preferred method for measuring BP in the ward, and these devices were validated according to standardized conditions and protocols [18]. The patient’s arm was supported, and clothing at the site of the cuff was removed. Cuff size was appropriate for the individual’s arm circumference, and the cuff was positioned at heart level (mi sternum). Patient BP was measured twice, and the arithmetic mean of two readings was recorded. Repeated measurements at 1-min interval after the 5-min rest period were initially conducted on both upper arms; when there was a difference in BP between arms, the arm with the higher BP values was used for all subsequent measurements.

According to the World Health Organization (WHO) and the International Society of Hypertension (ISH), the diagnostic criteria for hypertension were as follows: BP readings less than 140/90 mmHg are recognized to be normal; hypertension is defined as SBP greater than or equal to 140 mmHg and diastolic BP (DBP) greater than or equal to 90 mmHg or the use of any antihypertensive medications [19].

For all participants, an average SBP was determined for each 6-month block during follow-up; the average SBP of every 6-month period is represented by the time-averaged SBP (TA-SBP) [20]. TA-SBP was categorized as TA-SBP <125, 125–139, 140–149, and ≥150 mmHg. Similar to SBP, time-averaged values were determined for diastolic blood pressure (TA-DBP), blood uric acid (TA-UA), fasting plasma glucose (TA-glu), cholesterol (TA-CHOL), triglycerides (TA-TG), and LDL (TA-LDL), representing an average of all measurements collected every six months.

### Definition of CKD

Data about sex, race, age and sCr were used to calculate eGFR according to the CKD Epidemiology Collaboration (CKD-EPI) creatinine equation for each participant.

In detail, we defined eGFR (ml/min per 1.73 m^2^) according to the following: [21] for females with sCr ≤ 0.7 mg/dL (62 µmol/L), 144 × (sCr/0.7) − 0.329 × 0.993 × age; for females with sCr > 0.7 mg/dL (62 µmol/L), 144 × (sCr/0.7) − 1.209 × 0.993 × age; for males with sCr ≤ 0.9 mg/dL (80 µmol/L), 141 × (sCr/0.9) − 0.411 × 0.993 × age; and for males with sCr > 0.9 mg/dL (80 µmol/L), 141 × (sCr/0.9) − 1.209 × 0.993 × age. The sCr value used in the equation was the baseline sCr value. CKD in our cohort was defined as the presence of eGFR < 60 mL/min per 1.73 m^2^ [4].

### Follow-up procedure and outcomes

All subjects were followed-up every month by telephone from study entry until the occurrence of stroke or death. The primary outcome of this study was the occurrence of stroke. Stroke was defined as the acute onset of focal neurologic or retinal symptoms associated with cerebral or retinal tissue ischemia.

Focal symptoms included hemiparesis, aphasia, visual field cuts, monocular blindness, diplopia, dysarthria, and postural imbalance. The diagnosis of stroke described above was based on the WHO criteria [22] and was made by a neurologist and confirmed on brain CT, brain MRI, or both. During follow-up, patients with subarachnoid hemorrhage, lacunar infarction, TIA or cardiogenic embolism were also excluded from this study.

### Statistical analyses

All data were crosschecked by two physicians for completeness and then entered into the database with Epi Data 2.0 by trained personnel. Data were analyzed using Microsoft Excel (Redmond, WA) and SAS version 9.2 (SAS Institute Inc., Cary, NC, USA). Continuous variables are expressed as the mean ± standard deviation (SD) and were compared using a *t*-test. Categorical variables are presented as proportions and were compared using a χ^2^ test. All *P* values were two-tailed; a *P* value <0.05 was considered statistically significant.

First, we stratified the history of hypertensive diseases into three strata (class 1, class 2, class 3, based on JNC8) and performed a χ^2^-test between every two strata. Another χ^2^-test was performed to identify the risk associated with strata of different hypertensive disease histories, both in participants with and without CKD. We subsequently tested the effects of CKD and TA-SBP on stroke risk in univariate analysis, parsimonious models including only CKD and TA-BP, and fully adjusted Cox proportional hazards models. Variables with *p* < 0.1 and the well-established predictors were selected as confounding variables in the multivariable analyses. Then, we stratified TA-SBP into four predetermined strata (<125, 125 to 139, 140 to 149, and ≥ 150 mm Hg) and performed survival analyses using Kaplan–Meier techniques to estimate the risk of stroke associated with different SBP strata in participants with and without CKD.

## Results

A total of 665 patients with an average age of 87.01 ± 6.96 years were included in this study, and 77.7% were male and 22.3% were female. Among the 665 patients, 449 (67.5%) had an eGFR higher than or equal to 60 mL/min/1.73 m^2^, and 216 (32.5%) were diagnosed with CKD. Of those with CKD, the prevalence of stage 3 (eGFR 30–59 mL/min/1.73 m^2^) was 93.5%, while only 6.5% of subjects had an eGFR lower than 30 mL/min/1.73 m^2^ (stages 4 and 5 CKD). All individuals had hypertension, and 549 (82.4%) were on antihypertensive medications. Individuals with CKD were more likely to be on antihypertensive medications than those without CKD.

After a median follow-up of 111 months, 190 (28.6%) participants developed stroke, including 25 (3.8%) hemorrhagic strokes. Data for these participants, including basic demographics, medical history and laboratory data according to the presence or absence of CKD, are provided in Table 1.

**Table 1.** Baseline demographics and laboratory data by renal function status in elderly patients.

Variables	CKD(*n* = 216)	Non-CKD(*n* = 449)	*P*
Demographics			
Age (years)	87.01 ± 6.96	82.93 ± 7.31	<0.001
60–69 (years)	2(0.90%)	19(4.20%)	
70–79 (years)	28(13.00%)	124(27.60%)	
80–89 (years)	102(47.20%)	226(50.30%)	
>90 (years)	84(38.9%)	80(17.80%)	
Female gender	54(25.00%)	94(20.94%)	0.238
Medical history			
Diabetes mellitus	92(42.59%)	180(40.09%)	0.539
Hyperlipidemia	69(31.94%)	167(37.19%)	0.185
Coronary heart disease	144(66.67%)	269(59.91%)	0.093
Hyperuricemia	31(14.35%)	47(10.47%)	0.145
Malignant tumors	40(18.52%)	57(12.69%)	0.046
Cigarette use	58(26.85%)	139(30.96%)	0.278
Alcohol use	19(8.80%)	76(16.93%)	0.005
Antihypertensive drug use	187(86.57%)	361(80.40%)	0.050
Physical examination			
BMI (kg/m^2^)	23.90 ± 3.22	24.08 ± 3.22	0.517
TA-SBP (mm Hg)	133.95 ± 10.46	132.46 ± 10.00	0.075
TA-DBP (mm Hg)	73.60 ± 4.75	74.98 ± 5.75	0.002
Laboratory data			
eGFR (Baseline, ml/min/1.73 m^2^)	48.86 ± 10.33	74.74 ± 10.18	<0.001
TA-blood uric acid (mmol/L)	345.84 ± 74.54	315.27 ± 69.98	0.395
TA-low density lipoprotein (mmol/L)	2.75 ± 0.60	2.78 ± 0.68	0.664
TA-high density lipoprotein (mmol/L)	1.12 ± 0.29	1.11 ± 0.30	0.947
TA-triglyceride (mmol/L)	1.38 ± 0.63	1.43 ± 0.71	0.347
TA-fasting plasma glucose (mmol/L)	5.69 ± 1.43	5.87 ± 1.58	0.134
Outcomes			
Stroke (excluding lacunar infarction)	64(29.62%)	126(28.06%)	0.675
Ischemic stroke	55(25.46%)	109(24.28%)	0.740
Hemorrhagic stroke	9(4.17%)	16(3.56%)	0.702
Mortality	16(7.40%)	31(6.90%)	0.813

Categorical variables are presented as *n* (%) and continuous variables as mean ± SD.

SBP: systolic blood pressure; DBP: diastolic blood pressure; TA: time-averaged (mean of all values acquired in a six-month period).

**Figure 2.**
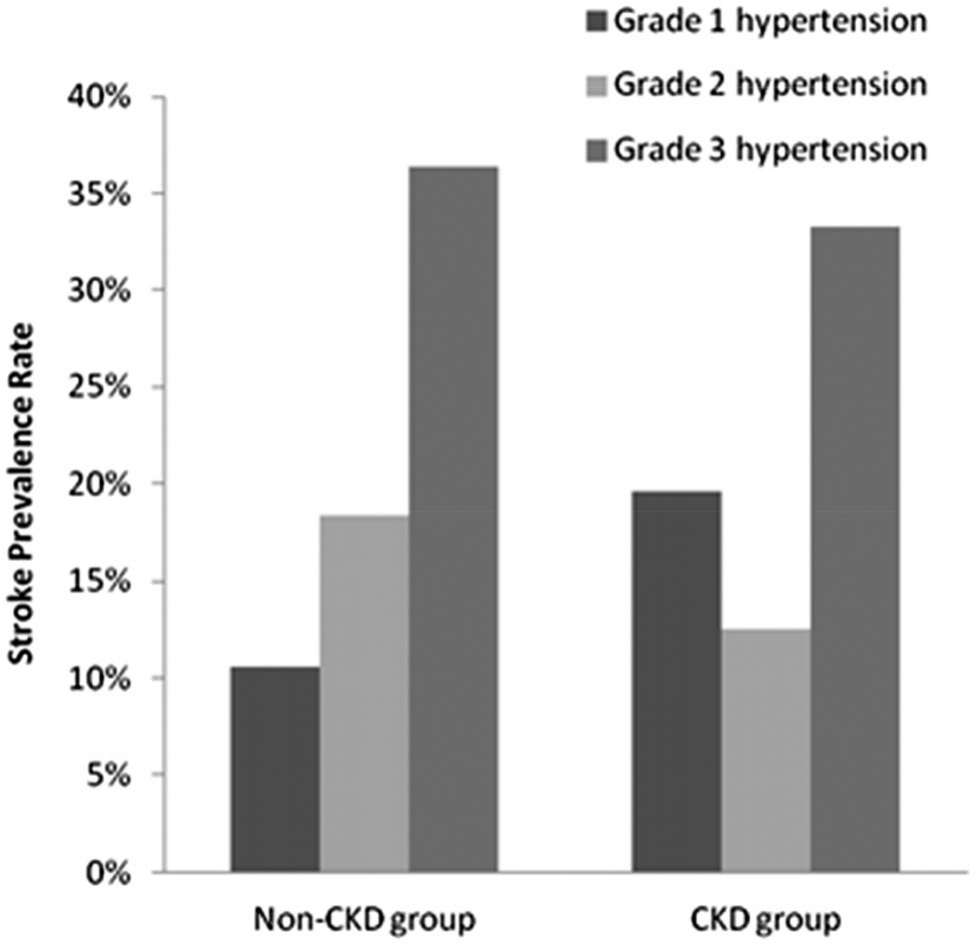
The relationship between the severity of hypertension and the prevalence of stroke in elderly patients with and without chronic kidney disease (CKD).

We then determined the relationship between the medical history of hypertension, baseline CKD status, BP levels, and the risk of developing stroke. In hypertensive individuals without CKD, the incidence of stroke differed significantly based on baseline BP (*p* < 0.001). Among patients with grade 3 hypertension, the incidence of stroke was significantly higher than among those with other grades of hypertension (*p* < 0.001). However, no statistically significant difference was observed in CKD patients (Figure 2). A logistic regression showed that TA-SBP was the most significant predictor of stroke among elderly patients in this study. Among all participants, there was a 28% increase in the risk of stroke for every 10 mmHg increase in TA-SBP (relative risk [RR] 1.028, 95% confidence interval [CI] 1.011–1.044), independent of TA-DBP. For CKD and non-CKD patients, there were 19% (RR 1.0195, 95% CI 0.991–1.048) and 32% (RR 1.032, 95% CI 1.001–1.054) increases in the risk of stroke for every 10 mm Hg increase in TA-SBP, respectively (Tables 2–4).

**Table 2.** Univariate and multiple logistic regression models (All patients).

Variables	Un-adjusted			Adjusted		
	OR	95%CI of OR	*P*	OR	95%CI of OR	*P*
CKD	1.079	(0.755,1.543)	0.675			
Age (years)	1.034	(1.011,1.059)	0.005	1.038	(1.011,1.065)	0.005
Female gender	0.658	(0.428,1.012)	0.057	0.635	(0.408,0.990)	0.045
Diabetes mellitus	1.040	(0.739,1.464)	0.822			
Hyperlipidemia	1.019	(0.717,1.447)	0.918			
Coronary heart disease	1.099	(0.775,1.558)	0.596			
Hyperuricemia	1.052	(0.626,1.767))	0.848			
Malignant tumors	0.608	(0.360,1.027)	0.063	0.606	(0.354,1.036)	0.067
Cigarette use	1.306	(0.910,1.875)	0.148			
Alcohol use	1.116	(0.695,1.792)	0.649			
Antihypertensive drug use	1.076	(0.689,1.682)	0.747			
BMI	0.952	(0.903, 1.004)	0.072	0.962	(0.910,1.017)	0.175
TA-SBP	1.028	(1.011,1.045)	0.001	1.032	(0.991,1.075)	0.087
TA-DBP	1.036	(1.003,1.069)	0.031	1.018	(0.997,1.039)	0.1225
TA-eGFR	0.998	(0.987,1.008)	0.688			
TA-blood uric acid	1.000	(0.998,1.002)	0.943			
TA-low density lipoprotein	1.108	(0.837,1.468)	0.473			
TA-high density lipoprotein	1.054	(0.596,1.863)	0.857			
TA-triglyceride	0.969	(0.755,1.243)	0.804			
TA-fasting plasma glucose	1.032	(0.928,1.149)	0.559			

**Table 3.** Univariate and multiple logistic regression models (non-CKD).

Variables	Un-adjusted			Adjusted		
	OR	95%CI of OR	*P*	OR	95%CI of OR	*P*
Age (years)	1.035	(1.005,1.066)	0.020	1.034	(1.002,1.067)	0.038
Female gender	0.737	(0.434,1.252)	0.260			
Diabetes mellitus	1.071	(0.704,1.628)	0.749			
Hyperlipidemia	0.960	(0.626,1.471)	0.851			
Coronary heart disease	1.024	(0.672,1.559)	0.913			
Hyperuricemia	0.763	(0.375,1.550)	0.454			
Malignant tumors	0.904	(0.482,1.696)	0.754			
Cigarette use	1.288	(0.831,1.995)	0.257			
Alcohol use	0.900	(0.515,1.572)	0.710			
Antihypertensive drug use	0.854	(0.513,1.420)	0.542			
BMI	0.948	(0.889,1.012)	0.108			
TA-SBP	1.032	(1.001,1.054)	0.003	1.020	(0.995,1.047)	0.116
TA-DBP	1.043	(1.004,1.083)	0.030	1.035	(0.987,1.085)	0.156
TA-eGFR	1.006	(0.986,1.026)	0.571			
TA-blood uric acid	0.998	(0.995,1.001)	0.264			
TA-low density lipoprotein	1.234	(0.887, 1.717)	0.212			
TA-high density lipoprotein	1.789	(0.908,3.525)	0.092	1.737	(0.859,3.512)	0.124
TA-triglyceride	0.949	(0.704,1.278)	0.729			
TA-fasting plasma glucose	1.029	(0.905,1.170)	0.664			

**Table 4.** Univariate and multiple logistic regression models (CKD).

Variables	Un-adjusted			Adjusted		
	OR	95%CI of OR	*P*	OR	95%CI of OR	*P*
Age (years)	1.037	(0.992,1.083)	0.109	1.039	(0.993,1.087)	0.102
Female gender	0.526	(0.251,1.102)	0.089	0.638	(0.286,1.424)	0.273
Diabetes mellitus	0.977	(0.541,1.764)	0.938			
Hyperlipidemia	1.170	(0.630,2.175)	0.619			
Coronary heart disease	1.268	(0.674,2.384)	0.461			
Hyperuricemia	1.616	(0.733,3.562)	0.234			
Malignant tumors	0.283	(0.106,0.761)	0.012	0.290	(0.106,0.791)	0.016
Cigarette use	1.364	(0.716,2.595)	0.345			
Alcohol use	2.324	(0.896,6.026)	0.083	1.599	(0.587,4.352)	0.358
Antihypertensive drug use	2.212	(0.804,6.085)	0.124			
BMI	0.962	(0.876,1.056)	0.412			
TA-SBP	1.019	(0.991,1.048)	0.196			
TA-DBP	1.022	(0.961,1.087)	0.480			
TA-eGFR	0.986	(0.960,1.013)	0.316			
TA-blood uric acid	1.003	(0.999,1.007)	0.160			
TA-low density lipoprotein	0.835	(0.481,1.449)	0.522			
TA-high density lipoprotein	0.281	(0.088,0.896)	0.032	0.400	(0.114,1.410)	0.154
TA-triglyceride	1.026	(0.648,1.625)	0.913			
TA-fasting plasma glucose	1.046	(0.859,1.273)	0.653			

**Figure 3.**
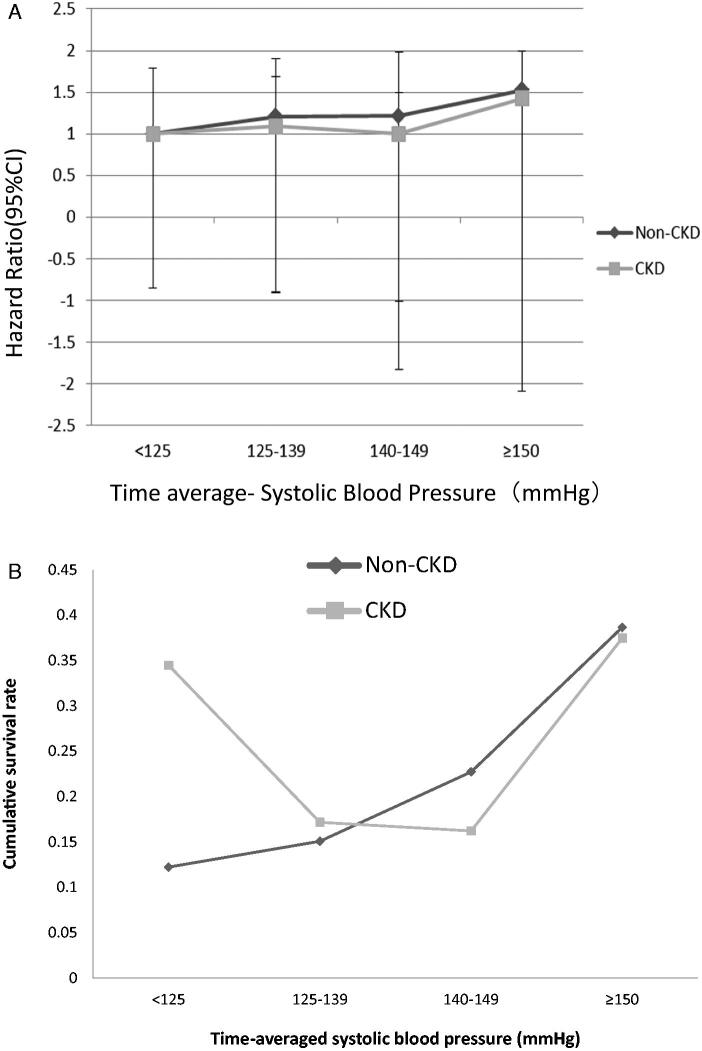
(A) The hazard ratio of stroke across different groups. (B) The cumulative survival rate for risk of stroke according to different groups.

For hypertensive patients, the risk of stroke associated with each of the four TA-SBP groups (based on posttreatment levels) is estimated in Table 5 and Figure 3(A). Kaplan–Meier survival analysis was performed for each group in each stratum to derive the median cumulative survival rate for each blood pressure stratum, as shown in Supplementary Materials Table 1 and Figure 3(B). For non-CKD patients, the cumulative survival rate of patients who had a stroke increased with increasing systolic blood pressure levels (*p* < 0.05). However, for patients with CKD, a J-shaped relationship was observed for the cumulative stroke survival rate across the entire TA-SBP spectrum (Figure 3(B)). For those with a TA-SBP less than 125 mmHg, the cumulative survival rate of stroke increased with decreasing TA-SBP levels (*p* < 0.05). For CKD patients with a TA-SBP level between 125 and 149 mmHg, no significant difference in the cumulative survival rate of stroke was observed (*p* > 0.05). Finally, for those with a TA-SBP greater than 150 mm Hg, the cumulative survival rate of stroke increased with higher TA-SBP levels (*p* < 0.01). This J-shaped relationship was not found in participants without CKD.

**Table 5.** Hazard ratios (95% CI) for stroke stratified by of SBP.

	SBP(mm Hg)				
	<125	125–139	140–149	≥150	*P* for trend^#^
All^a^	1	1.200(0.845,1.704)	1.169(0.746,1.833)	1.297(0.740,2.273)	0.106
Non-CKD^b^	1	1.210(0.791,1.852)	1.213(0.693,2.122)	1.528(0.767,3.044)	0.241
CKD^c^	1	1.092(0.600,1.987)	0.999(0.496,2.011)	1.422(0.575,3.516)	0.623

^a^adjusted by age, sex, malignant tumors, BMI and TA-DBP.

^b^adjusted by age, TA-high density lipoprotein and TA-DBP.

^c^adjusted by age, sex, malignant tumors, alcohol use and TA-high-density lipoprotein.

^#^a trend test was performed after the median value of each quintile was entered into the model and treated as a continuous variable.

## Discussion

As our population is growing older, health care for the elderly population emerges as an important issue. Age itself can be an independent risk factor for the occurrence of CKD [3,23] A cross-sectional study using a representative sample of the US population showed that approximately 3% (5.6 million) of civilians had elevated sCr levels, and 70% of these individuals were hypertensive [24]. Both previous studies [25] and our findings suggest that hypertension is more difficult to control in CKD patients. More than 50% of patients with CKD require three or more medications to control their BP. CKD increases BP through several mechanisms, including impaired sodium excretion and premature vascular aging, which subsequently reduce baroreceptor sensitivity, increase sympathetic nervous tone, and activate the renin–angiotensin–aldosterone system. Underlying intrinsic kidney diseases such as glomerulonephritis can also cause hypertension [26]. Other studies also identified an association between reduced eGFR and hypertension [24], while subsequent reports suggested that a sustained increase in BP is the most important risk factor for developing stroke [5]. Therefore, BP control could be an important approach for reducing the risk of stroke in elderly CKD patients.

In the general population, studies have shown that the lower the BP is, the greater the benefit of BP control, especially among CKD patients [27,28]. However, there are no studies on older elderly individuals. Elderly patients with CKD are different from their younger counterparts, and the average age of the study subjects in our study was 87.01 ± 6.96 years old. The literature indicates a J-curve relationship between BP and the risk of cardiovascular events, but such a relationship between BP and the risk of stroke is seldom addressed [29]. Several studies have investigated the association between SBP levels and the HR of stroke in CKD patients [13]. One of these studies reported that among individuals with CKD, those with SBP below 120 mmHg had a significantly higher risk of stroke than those with SBP levels between 120- and 129-mm Hg (HR 2.51, 95% CI 1.30 to 4.87) [4]. The risk increased further for those with CKD and BP levels higher than 130 mmHg, but the same relationship between risk and SBP was not observed in individuals without CKD [14]. However, few studies have specifically examined the relationship between BP levels and the risk of stroke in a large CKD cohort consisting of elderly patients.

In the current study, we discovered a significant association between a history of hypertension and an elevated risk of stroke, regardless of baseline renal function. Among non-CKD patients, the relationship between baseline BP levels and the risk of stroke was statistically significant. The risk of stroke exhibited minor differences between patients with stage 1 and 2 hypertensions, but the risk associated with stage 3 hypertension was significantly higher than that associated with stage 1 and 2 hypertensions (*p* < 0.05). For CKD patients, there was no difference in the risk of stroke among patients with different baseline BP levels.

SBP is a well-known risk factor for stroke in the general population [30]. We used the concept of TA-SBP to accommodate fluctuations in BP during the follow-up period to address whether patients with initially higher BP have the same prognosis as those with initially lower BP if both groups of patients achieve target BP after treatment. In addition to baseline BP, we found that TA-SBP was also an independent risk factor for incident stroke in patients with and without CKD; however, those with CKD and the lowest BP levels still had a higher risk for incident stroke than those with SBP between 120 and 129 mmHg. For patients without CKD, the risk of stroke increased linearly as SBP rose among all BP levels analyzed. This discrepancy may reflect an imbalance in the therapeutic intensity for hypertension between individuals with and without CKD.

Our cohort of elderly CKD patients consisted predominantly of stage-3 patients (95.4%), implying that the influence of CKD on stroke emerges at an early stage. One plausible reason could be that changes in BP had already started in the very early stage of CKD. Elderly patients enduring a prolonged period of hypertension tend to contract multimorbidity and develop arteriosclerosis; when their BP levels decrease with treatment, cerebral blood flow is significantly compromised, creating a tendency toward manifesting cerebrovascular disease [30]. Another reason for the difference in the observed relationships in CKD and non-CKD patients could be interference by other comorbid conditions, including cardiomyopathy and malnutrition. Patients who are weaker are also more likely to exhibit lower BP and cholesterol levels [31]. In our research, discrepancies may be caused by other comorbid conditions, such as patients with CKD having a higher history of malignancy, higher use of antihypertensive drugs, lower diastolic blood pressure and alcohol consumption (Table 1). In light of this, lower BP levels might be a surrogate for poorer overall health status and an increased burden of cardiovascular diseases. Therefore, lower BP levels may be a token of the associated higher risk of stroke, instead of the cause of stroke [4].

This study has several strengths. First, the number of participants was large, and the ethnicity of the participants was different from that in other studies. Additionally, the comprehensive patient evaluation (clinical and laboratory tests) and long follow-up duration also lends support to our findings. However, several limitations should be noted. The eGFR obtained by the CKD-EPI equation might not be accurate. The proteinuria status in these patients was not routinely recorded; thus, we could not account for this factor. The influence of proteinuria on the risk of stroke in this cohort could not be evaluated. In addition, the predominance of stage-3 CKD patients in our cohort precludes extrapolation of our findings to those with more advanced CKD. Measurement of home BP allows for the identification of patients with white-coat and masked hypertension, the latter of which is more prevalent in patients with reduced kidney function and is associated with target-organ damage and adverse outcomes [18]. However, we did not include home BPs. Additionally, we did not include the class of drugs that were used in the analysis as covariates. It is possible that different drugs are used in chronic kidney disease patients that in those who do not have chronic kidney disease, and the drugs could be mediators of this relationship. More data on drugs need to be collected in future work. Thus, further large-scale prospective studies are still needed to validate our findings.

## Conclusion

Elevated TA-SBP was an independent risk factor for stroke in individuals with and without CKD. There was a J-shaped relationship between SBP levels and the risk of stroke in CKD patients, and individuals with the lowest BP levels were at increased risk for developing stroke. This phenomenon was not found in the general population.

## Ethics approval and consent to participate

This study was performed in accordance with the principles of the Declaration of Helsinki. Informed consent was obtained from all individual participants enrolled in the study.

## Author contributions

FC participated in the study design, collected and analyzed the data, and wrote the manuscript, which was supervised by CHP. ZDL participated in the preparation, creation and presentation of the published work, specifically critical review, commentary and revision. ZH participated in data collection and data analysis. All authors read and approved the final manuscript.

## Supplementary Material

Supplemental Material

## Data Availability

The datasets generated and/or analyzed during the current study are available from the corresponding author on reasonable request.
