# Supplementary material for: Timed-averaged blood pressure showed a J-curve association with stroke in elderly chronic kidney disease patients
Source: Ren Fail. 2022 Oct 12;44(1):1677–86. doi: 10.1080/0886022X.2022.2131574 (PMC9578479; doi:10.1080/0886022X.2022.2131574)
Supplement: Supplemental Material [file IRNF_A_2131574_SM3606.pdf]

Supplementary Materials Table 1. Median and quartiles of the cumulative survival rate of stroke in patients

Variables	TA-SPB（mmHg）	Median	P25	P75
Non-CKD	＜125	0.12	0.07	0.22
	125-139	0.15	0.07	0.27
	140-149	0.23	0.10	0.37
	＞=150	0.39	0.16	0.87
CKD	＜125	0.34	0.08	0.68
	125-139	0.17	0.08	0.37
	140-149	0.16	0.07	0.31
	＞=150	0.37	0.15	0.71
